# Type 2 cytokines and scleroderma interstitial lung disease

**DOI:** 10.1007/s10238-023-01125-x

**Published:** 2023-07-01

**Authors:** Chiara Pellicano, Lorenzo Vantaggio, Amalia Colalillo, Krizia Pocino, Valerio Basile, Mariapaola Marino, Umberto Basile, Edoardo Rosato

**Affiliations:** 1 Department of Translational and Precision Medicine, Sapienza University of Rome, Viale Dell’Università 37, 00185 Rome, Italy; 2 UOC of Clinical Pathology, General Hospital San Pietro Fatebenefratelli, 00189 Rome, Italy; 3 Clinical Pathology Unit and Cancer Biobank, Department of Research and Advanced Technologies, IRCCS Regina Elena National Cancer Institute, 00144 Rome, Italy; 4 Department of Translational Medicine and Surgery, Università Cattolica del Sacro Cuore, Fondazione Policlinico A. Gemelli IRCCS, 00168 Rome, Italy; 5 UOC Clinical Pathology DEA II Level, Hospital Santa Maria Goretti-ASL Latina, 04100 Latina, Italy

**Keywords:** Systemic sclerosis, Interstitial lung disease, Th2 cytokines, Ground glass, CALIPER

## Abstract

Interstitial lung disease (ILD) is a life-threatening complication of systemic sclerosis (SSc). Type 2 (Th2) cytokines play a pivotal role in airway disease. Study aim was to evaluate serum level of Th2 interleukin (IL) and chemokine in SSc-ILD. Serum levels of IL-4, IL-5, IL-11, IL-13, IL-21, IL-31 and CXCL-13 were measured by Bio-Plex Multiplex Immunoassays in 60 SSc patients and 20 healthy controls (HC). Pulmonary function tests with diffusion lung capacity for carbon monoxide (DLco) and high resolution computed tomography (HRCT) were performed in SSc patients. ILD is defined as fibrotic changes (ground glass, reticular and honeycombing), assessed by Computer-Aided Lung Informatics for Pathology Evaluation and Ratings (CALIPER) software, affecting at least 10% of the lungs. Serum levels of Th2 cytokines were higher in SSc patients than HC. A linear correlation was observed between ground glass and IL-13 (*r* = 0.342, *p* < 0.01), IL-21 (*r* = 0.345, *p* < 0.01), IL-31 (*r* = 0.473, *p* < 0.001), IL-4 (*r* = 0.863, *p* < 0.001), IL-5 (*r* = 0.249, *p* < 0.05) and peripheral blood eosinophils (*r* = 0.463, *p* < 0.001). We found a negative correlation between DLco and IL-4 (*r* =  − 0.511, *p* < 0.001) and peripheral blood eosinophils (*r* =  − 0.446, *p* < 0.001). In the logistic regression analysis, IL-4 is associated with DLco ≤ 60% of the predicted [OR 1.039 (CI 95%: 1.015–1.064), *p* < 0.001], whilst mRSS [OR 1.138 (CI 95%: 1.023–1.266), *p* < 0.05] and IL-4 [OR 1.017 (CI 95%: 1–1.034), *p* < 0.05] were associated with ILD. Th2 inflammation could play a key role in early phase of SSc-ILD.

## Introduction

Systemic sclerosis (SSc) is an autoimmune disease characterized by immune system dysregulation, endothelial dysfunction and fibrosis of skin and internal organs. Interstitial lung disease (ILD) is common in SSc and about 80% of patients will develop ILD during the disease course [1]. Although most patients have a stable or slowly progressive disease, showing a slow decline in lung function or a minimal increase in the extent of pulmonary fibrosis by high resolution computed tomography (HRCT), 25–30% of them will ultimately progress to respiratory failure or death [2]. Of the SSc-related deaths, 35% were attributed to ILD [3].

The underlying pathophysiologic mechanisms of SSc-ILD is still not completely understood [2]. The pathogenesis of SSc-ILD is initially characterized by an injury to alveolar epithelial and vascular endothelial cells which promotes the activation of the immune system and the recruitment of inflammatory cells and the production of profibrotic mediators, in the attempt to repair the damage, with accumulation of myofibroblasts [4, 5]. Infiltration by inflammatory T cells is thought to precede the development of lung fibrosis and in this early inflammatory phase, profibrotic type 2 (Th2) cytokines, such as IL-4, IL-5, IL-13, play a key role [6, 7]. Indeed, an abnormal profibrotic Th2-polarized T cell response has been postulated to mediate tissue damage and fibrosis in SSc-ILD as Th2 cytokines lead to the activation of alternative inflammatory pathways and to the transcription of transforming growth factor (TGF)-β, involved in induction and progression of fibrosis [8, 9]. Moreover, it has been demonstrated that IL-4/IL-13 axis upregulate genes known to be involved in the mechanisms of wound healing and fibrosis in murine models and in vitro, influencing the activation of myofibroblasts [10].

Th2 cytokines also contribute to the differentiation and migration of eosinophils, which are involved in systemic inflammatory process in SSc patients [11–13]. In the earlier course of ILD, pulmonary function tests (PFTs) show a reduction of the diffusion lung capacity for carbon monoxide (DLco) with preserved forced vital capacity (FVC) and HRCT shows a ground glass pattern with typical subpleural and posterobasal distribution [14]. At a later stage, fibroblasts proliferation and activation lead to extracellular matrix deposition and fibrosis of the lung parenchyma. PFTs show a restrictive pattern with a further decline of DLco and a reduction of FVC [14]. HRCT shows a reticular pattern with intralobular opacity and thickening of the interlobular septa and, eventually, a honeycombing pattern with traction bronchiectasis [14]. Aim of the study was to evaluate the Th2 cytokines serum levels in SSc-ILD.

## Materials and methods

### Subjects

Sixty SSc patients, fulfilling the American College of Rheumatology/European League Against Rheumatism Collaborative Criteria (2013 ACR/EULAR) for SSc [15] and twenty healthy controls (HC), matched for sex and age and recruited among healthcare workers, were enrolled in this study.

Cardiopulmonary diseases not related to SSc, pulmonary arterial hypertension (PAH), heart and hepatic failure, end stage renal diseases, malignancies, allergic diseases were exclusion criteria. Smokers, pregnant or breastfeeding women and patients treated in the last 6 months with immunosuppressive agents and corticosteroids at an equivalent dose of prednisone ≥ 10 mg/day were also excluded.

The study was conducted according to the Declaration of Helsinki and all participants provided written informed consent. The study was approved by the ethics committee of Sapienza University (IRB 0304).

### Clinical assessment

The modified Rodnan skin score (mRSS) was used to assess skin involvement and the disease subset was assessed according to LeRoy et al. [16] Disease activity and disease severity were measured by the disease activity index (DAI) [17] and disease severity scale (DSS) [18], respectively.

### Nailfold videocapillaroscopy

Nailfold videocapillaroscopy (NVC) was performed at the level of the distal phalanx of the second, third and fourth fingers of both hands using a videocapillaroscope equipped with a 500 × magnification lens (Pinnacle Studio Version 8 software, Corel, Ottawa, Canada). According to Cutolo et al. [19], the capillaroscopic images were classified in *early*, *active*, and *late* pattern. The NVC was performed by the same experienced operator, blinded for laboratory assessment and for other clinical characteristics of SSc patients.

### Pulmonary function tests

PFTs parameters [forced expiratory volume in the 1st second (FEV1), FVC] and single-breath DLco were recorded by the same blinded experienced operator with a Quark PFT 2 spirometer (Cosmed, Rome, Italy) and expressed according to the standards recommended by the American/European Respiratory Society [20]. PFTs were performed at enrollment in this study.

### Chest high-resolution computed tomography

The HRCT performed during the study or in the previous 6 months were evaluated by the same expert radiologist. ILD was defined as fibrotic changes affecting at least 10% of the lung parenchyma according to previous study [21]. Radiological patterns (normal, ground glass, reticular, and honeycombing) were measured and expressed as percentage of total lung parenchyma through Computer-Aided Lung Informatics for Pathology Evaluation and Ratings (CALIPER) software by the same blinded radiologist [22].

### Laboratory assessment

Peripheral venous blood samples were collected in tubes and centrifuged at 3000 × *g* for 15 min at 19 °C. Serum samples were aliquoted into 1.5 mL Eppendorf tubes and stored at − 80 °C until the time of assay. The assessment of serum levels of IL-4, IL-5, IL-11, IL-13, IL-21, IL-31, CXCL-13 cytokines was carried out using a Bio-Plex Multiplex Immunoassays allow to simultaneously measure analytes available as ready-to-use premixed multiplex panels (BIO-RAD, Hercules, CA, USA). All blood tests were performed in a single analytical session, following the instructions provided by the manufacturers. Each sample was tested twice to minimize eventual discrepancies, and all tests were performed in the same laboratory with the same instrument by the same experienced operator, blinded of the clinical information of the handled sample.

### Statystical analysis

SPSS version 26.0 software (Bioz, Los Altos, CA) was used for statistical analysis. After an evaluation of normality by using the Shapiro–Wilk test, continuous variables were expressed as median and interquartile range (IQR). Student’s or Mann–Whitney’s *t*-test were performed to evaluate differences between groups. Differences between categorical variables were evaluated by the chi-square or Fisher’s exact test, as appropriate. The Pearson or Spearman correlation test was used for bivariate correlations. Stepwise logistic regression analysis was used to evaluate the association between a dependent dichotomic variable (ILD or DLco ≤ 60% of the predicted) and continuous [age (years), duration disease (years), mRSS, IL-4 (pg/ml)] or cathergorical [Scl70 (yes or no)] independent variables. Results were expressed as odds ratio (OR) and 95% confidence interval (95% CI). A *p*-value < 0.05 was considered significant.

## Results

Fifty-five (91.7%) patients were females. Median age was 56 years (IQR 49–63 years). Thirty-one (51.7%) patients had diffuse cutaneous SSc (dcSSc) and 29 (48.3%) had limited cutaneous SSc (lcSSc). Thirteen (21.7%) patients had DLco ≤ 60% of predicted and 19 (31.7%) patients had radiological ILD. Demographic and clinical features of SSc patients are shown in Table 1.

**Table 1:** Demographic and clinical features of systemic sclerosis (SSc) patients

Age, years, median and IQR	56 (49–63)
Female, *n* (%)	55 (91.7)
dcSSc, *n* (%)	31 (51.7)
Disease duration, years, median and IQR	11 (7–16)
mRSS, median and IQR	11 (8–16)
Autoantibodies	
Anti-topoisomerase I, *n* (%)	29 (48.3)
Anti-centromere, *n* (%)	16 (26.7)
Anti-RNApolimerase III, *n* (%)	2 (3.3)
ANA, *n* (%)	13 (21.7)
NVC	
Early, *n* (%)	9 (15)
Active, *n* (%)	18 (30)
Late, *n* (%)	33 (55)
DAI, median and IQR	2.3 (1.26–3.88)
DSS, median and IQR	7 (5–9)
sPAP, mmHg, median and IQR	28 (25–31)
DLco, % of predicted, median and IQR	74 (62–84) 13 (21.7)
DLco ≤ 60% of predicted, *n* (%)	
ILD,* n* (%)	19 (31.7)
Normal, median and IQR	91.83 (89.37–94.82)
Ground glass, median and IQR	2.6 (0.89–5.05)
Reticular, median and IQR	1.72 (1.3–2.27)
Honeycombing, median and IQR	2.41 (1.36–4.4)

*SSc* Systemic sclerosis; *dcSSc* diffuse cutaneous systemic sclerosis; *mRSS* modified rodnan skin score; *ANA* antinuclear antibodies; *NVC* Nailfold videocapillaroscopy; *DAI* disease activity index;* DSS* disease severity scale; *sPAP* Pulmonary arterial systolic pressure; *DLco* diffusing capacity of lung for carbon monoxide; *ILD* interstitial lung disease; *IQR *interquartile range

The median value of circulating eosinophils was 135/μl (IQR 65–195). Only 3 (5%) patients had peripheral blood eosinophils ≥ 300/μl whilst 29 (46.8%) patients had peripheral blood eosinophils ≥ 150/μl. The median value of serum IgE was 85 KU/l (IQR 61.50–99.5).

SSc patients had a statistically significant higher median value of Th2 cytokines and chemokine than HC (Table 2). Median CXCL-13 serum levels were significantly higher in SSc patients compared to HC [124.97 ng/ml (IQR 82.78–212.92) vs 76.55 ng/ml (IQR 48.58–105.18), *p* < 0.001]. Median IL-11 serum levels were significantly higher in SSc patients compared to HC [239.16 ng/ml (IQR 172.46–351.81) vs 33.14 ng/ml (IQR 26.49–98.25), *p* < 0.001]. Median IL-13 serum levels were significantly higher in SSc patients compared to HC [241.47 ng/ml (IQR 164.35–460.7) vs 68.54 ng/ml (IQR 48–103.6), *p* < 0.001]. Median IL-21 serum levels were significantly higher in SSc patients compared to HC [130.25 ng/ml (IQR 57.05–259.78) vs 17.52 ng/ml (IQR 14.83–27.13), *p* < 0.001]. Median IL-31 serum levels were significantly higher in SSc patients compared to HC [298.12 ng/ml (IQR 180.81–398.6) vs 55.71 ng/ml (IQR 41.98–83.06), *p* < 0.001]. Median IL-4 serum levels were significantly higher in SSc patients compared to HC [111.06 ng/ml (IQR 92.09–136.64) vs 16.68 ng/ml (IQR 11.68–29.37), *p* < 0.001]. Median IL-5 serum levels were significantly higher in SSc patients compared to HC [15.73 ng/ml (IQR 11.65–19.24) vs 5.93 ng/ml (IQR 4.46–6.61), *p* < 0.001].

**Table 2:** Comparative analysis of demographic features and serum levels of Th2 cytokines and chemokine between systemic sclerosis (SSc) patients and healthy controls (HC)

	SSc	HC	*p*
Age, years, median and IQR	56 (49–63)	54 (50–65)	> 0.05
Female, *n* (%)	55 (91.7)	18 (90)	> 0.05
IL-4, pg/ml, median and IQR	111.06 (92.09–136.64)	16.68 (11.68–29.37)	< 0.001
IL-5, pg/ml, median and IQR	15.73 (11.65–19.24)	5.93 (4.46–6.61)	< 0.001
IL-11, pg/ml, median and IQR	239.16 (172.46–351.81)	33.14 (26.49–98.25)	< 0.001
IL-13, pg/ml, median and IQR	241.47 (164.35–460.70)	68.54 (48–103.6)	< 0.001
IL-21, pg/ml, median and IQR	130.25 (57.05–259.78)	17.52 (14.83–27.13)	< 0.001
IL-31, pg/ml, median and IQR	298.12 (180.81–398.60)	55.71 (41.98–83.06)	< 0.001
CXCL-13, pg/ml, median and IQR	124.97 (82.78–212.92)	76.55 (48.58–105.18)	< 0.001

*SSc* Systemic sclerosis; *HC* Healthy controls; *IQR* Interquartile range

**Fig. 1:**
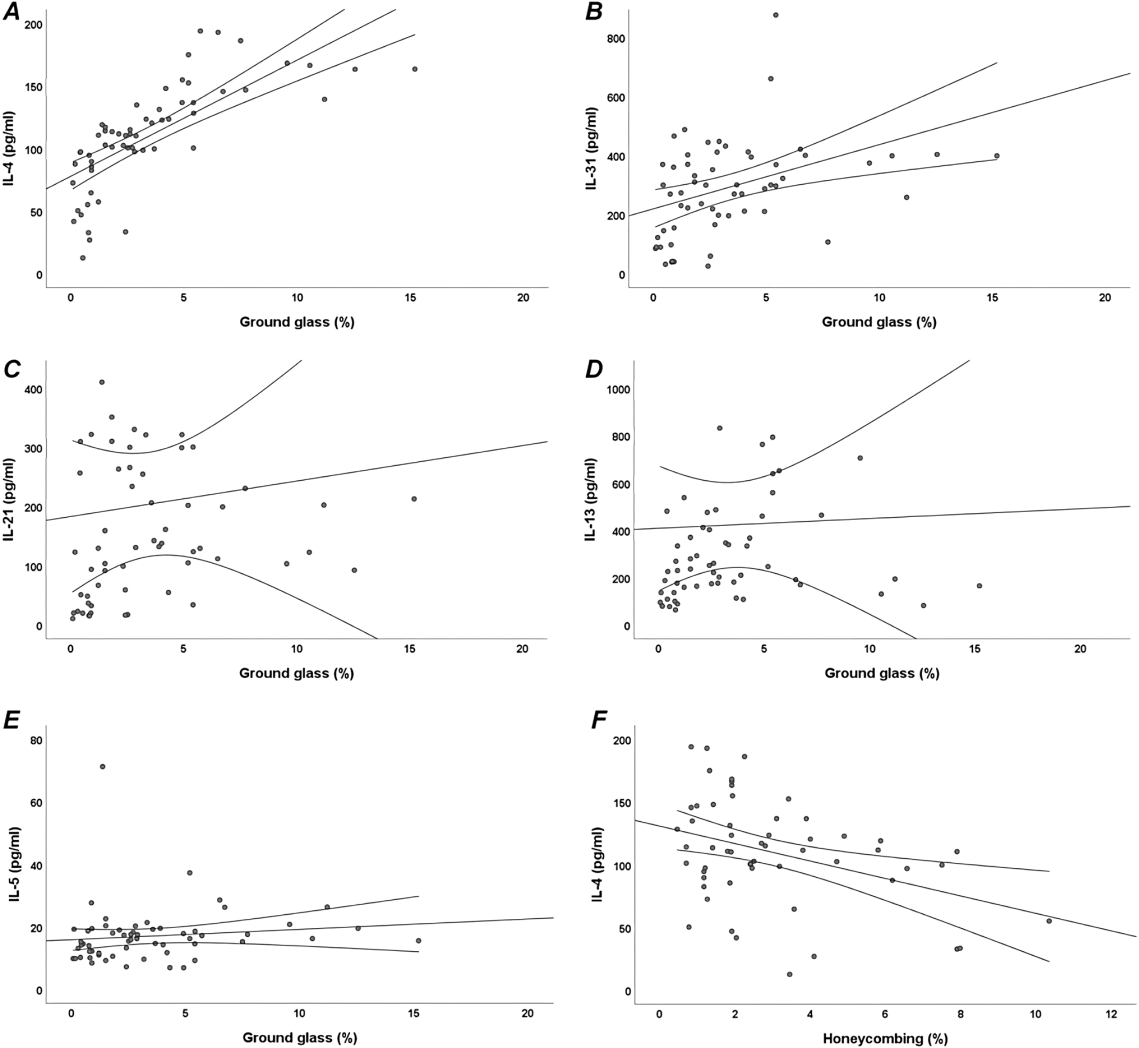
Bivariate correlations between Th2 cytokines serum levels and radiological patterns of interstitial lung disease (ILD), assessed through CALIPER software, in systemic sclerosis (SSc) patients. A: Positive linear correlation between IL-4 and ground glass (*r* = 0.863, *p* < 0.001); B: Positive linear correlation between IL-31 and ground glass (*r* = 0.473, *p* < 0.001); C: Positive linear correlation between IL-21 and ground glass (*r* = 0.345, *p* < 0.01); D: Positive linear correlation between IL-13 and ground glass (*r* = 0.342, *p* < 0.01); E: Positive linear correlation between IL-5 and ground glass (*r* = 0.249, *p* < 0.05); F: Negative linear correlation between IL-4 and honeycombing (*r* =  − 0.281, *p* < 0.05)

**Fig. 2:**
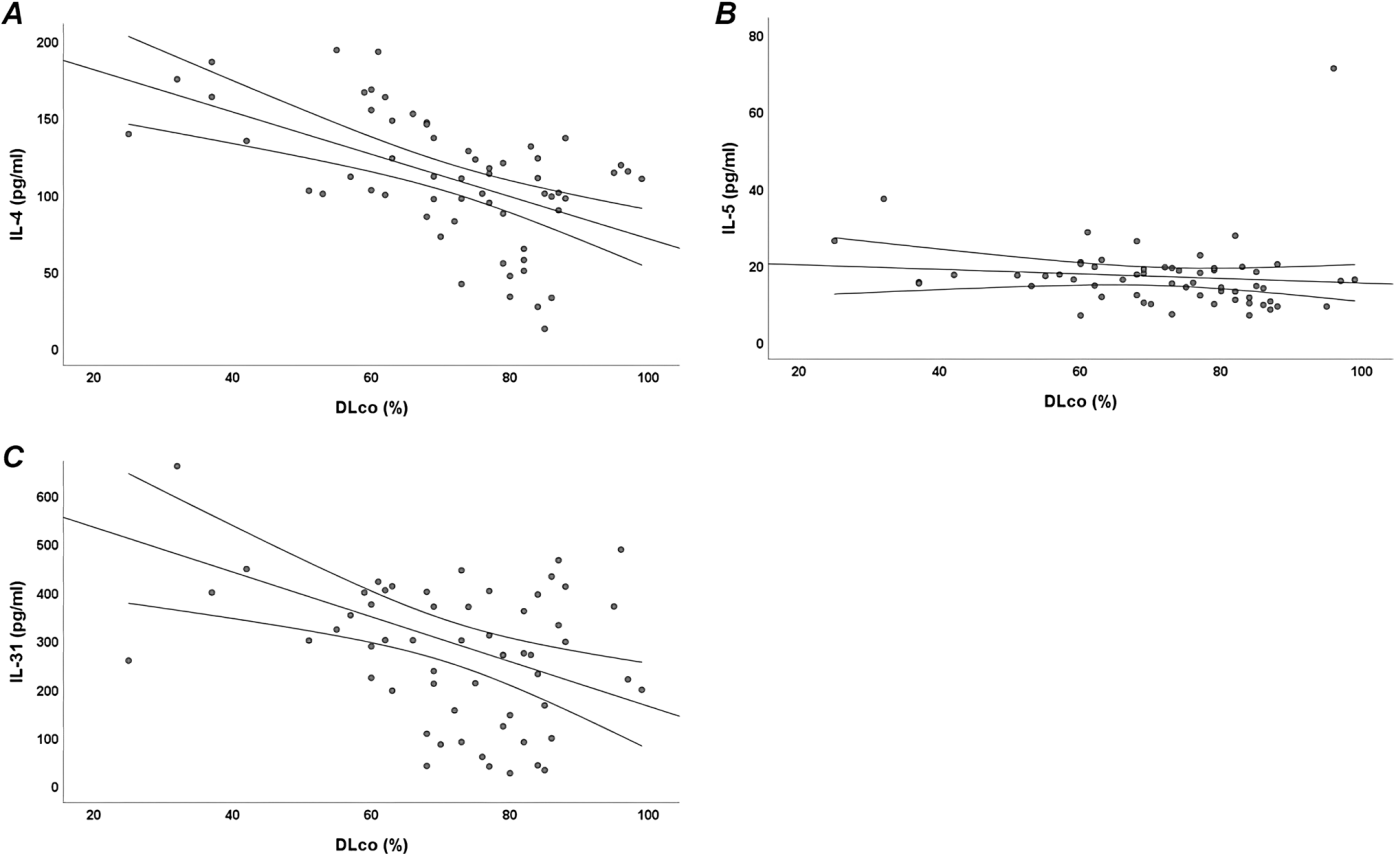
Bivariate correlations between Th2 cytokines serum levels and diffusing capacity of lung for carbon monoxide (DLco) in systemic sclerosis (SSc) patients. A: Negative linear correlation between IL-4 and DLco (*r* =  − 0.511, *p* < 0.001); B: Negative linear correlation between IL-5 and DLco (*r* =  − 0.259, *p* < 0.05); C: Negative linear correlation between IL-31 and DLco (*r* =  − 0.258, *p* < 0.05)

We found a statistically significant positive correlation between ground glass CALIPER pattern and IL-4 (*r* = 0.863, *p* < 0.001) and IL-31 (*r* = 0.473, *p* < 0.001) (Fig. 1a–b). Moreover, we showed a slightly statistically significant positive correlation between ground glass CALIPER pattern and IL-21 (*r* = 0.345, *p* < 0.01), IL-13 (*r* = 0.342, *p* < 0.01) and IL-5 (*r* = 0.249, *p* < 0.05) (Fig. 1c–e). A statistically significant negative correlation exists between normal CALIPER pattern and IL-4 (*r* =  − 0.414, *p* < 0.001). In addition, we found a slightly statistically significant negative correlation between normal CALIPER pattern and IL-31 (*r* =  − 0.289, *p* < 0.05) and IL-13 (*r* =  − 0.224, *p* < 0.05). Finally, we showed a statistically significant negative correlation between IL-4 and honeycombing CALIPER pattern (*r* =  − 0.281, *p* < 0.05) (Fig. 1f). We found a statistically significant negative correlation between IL-4 and DLco (*r* =  − 0.511, *p* < 0.001) (Fig. 2a). Moreover, we showed a slightly statistically significant negative correlation between DLco and IL-5 (*r* =  − 0.259, *p* < 0.05) and IL-31 (*r* =  − 0.258, *p* < 0.05) (Fig. 2b–c). No statistically significant correlations exist between CXCL-13 or IL-11 and DLco or CALIPER pattern.

**Fig. 3:**
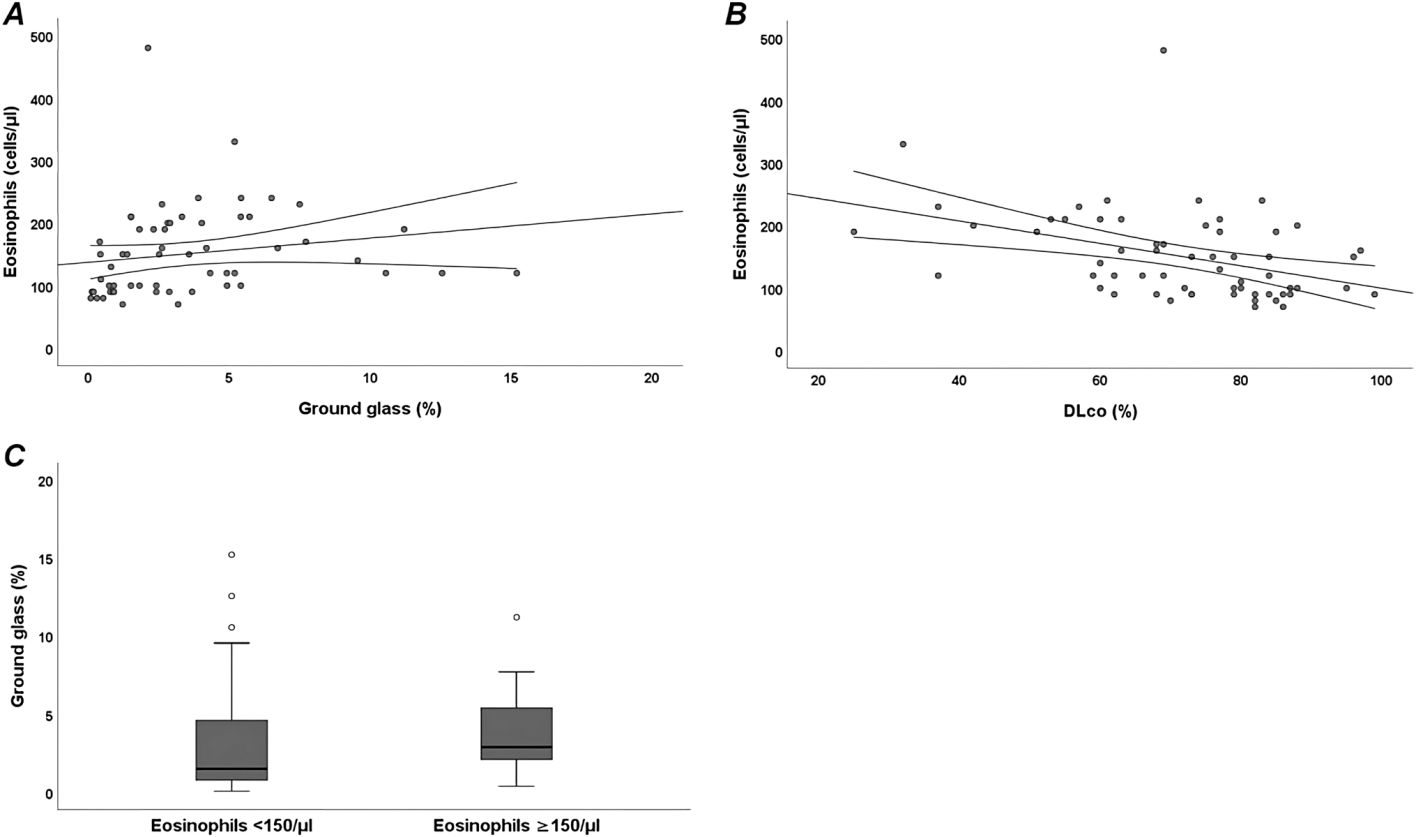
Peripheral blood eosinophil count in systemic sclerosis (SSc) patients and its association with variables of interstitial lung disease (ILD). A: Positive linear correlation between peripheral blood eosinophil count and ground glass CALIPER pattern (*r* = 0.463, *p* < 0.001); B: N egative linear correlation between peripheral blood eosinophil count and diffusing capacity of lung for carbon monoxide (DLco) (*r* =  − 0.446, *p* < 0.001); C: Comparative analysis of the percentage of ground glass parenchyma between systemic sclerosis (SSc) patients with peripheral blood eosinophils ≥ 150/μl and SSc patients with peripheral blood eosinophils < 150/μl (*p* < 0.05)

A statistically significant positive linear correlation was found between peripheral blood eosinophil count and IL-4 (*r* = 0.598, *p* < 0.001), IL-5 (*r* = 0.559, *p* < 0.001), IL-13 (*r* = 0.366, *p* < 0.01), IL-21 (*r* = 0.469, *p* < 0.001), IL-11 (*r* = 0.274, *p* < 0.05), IL-31 (*r* = 0.345, *p* < 0.01) and CXCL-13 (*r* = 0.309, *p* < 0.01). Moreover, we found a statistically significant positive correlation between peripheral blood eosinophil count and ground glass CALIPER pattern (*r* = 0.463, *p* < 0.001) (Fig. 3a) and a statistically significant negative correlation between peripheral blood eosinophil count and DLco (*r* =  − 0.446, *p* < 0.001) (Fig. 3b). The percentage of ground glass parenchyma was higher in SSc patients with peripheral blood eosinophils ≥ 150/μl compared to SSc patients with peripheral blood eosinophils < 150/μl [2.89% (IQR 2.10–5.40) vs 1.5% (IQR 0.76–4.90), *p* < 0.05] (Fig. 3c).

We did not find any statistically significant correlation between serum IgE and DLco (*r* = 0.144, *p* > 0.05) or ground glass CALIPER pattern (*r* =  − 0.03, *p* > 0.05).

The logistic regression analysis showed IL-4 as the only variable associated with DLco ≤ 60% of the predicted [OR 1.039 (CI 95%: 1.015–1.064), *p* < 0.001] (Table 3). The logistic regression analysis showed mRSS [OR 1.138 (CI 95%: 1.023–1.266), *p* < 0.05] and IL-4 [OR 1.017 (CI 95%: 1–1.034), *p* < 0.05] as variables associated with ILD (fibrotic involvement ≥ 10% of lung parenchyma) in SSc patients (Table 3).

**Table 3:** Logistic regression analysis showing the association between interstitial lung disease (ILD) or Diffusing Capacity of Lung for Carbon Monoxide (DLco) ≤ 60% of the predicted and independent variables

	DLco ≤ 60% of predicted		ILD	
	OR (CI 95%)	*p*	OR (CI 95%)	*p*
Age, years	1.040 (0.951–1.139)	> 0.05	1.029 (0.958–1.105)	> 0.05
Disease duration, years	0.956 (0.874–1.045)	> 0.05	0.973 (0.909–1.041)	> 0.05
mRSS	1.070 (0.952–1.203)	> 0.05	1.138 (1.023–1.266)	< 0.05
Scl70	1.493 (0.269–8.288)	> 0.05	0.288 (0.065–1.264)	> 0.05
IL-4, pg/ml	1.039 (1.015–1.064)	< 0.001	1.017 (1–1.034)	< 0.05

*mRSS* Modified Rodnan skin score; *DLco* diffusing capacity of lung for carbon monoxide; *ILD* interstitial lung disease; *OR* odds ratio; *CI* confidence interval

## Discussion

In this study, we demonstrated increased Th2 cytokines serum levels in SSc patients compared to HC. Moreover, we found a positive linear correlation between serum Th2 cytokines and the extent of ground glass pattern assessed by CALIPER software in SSc patients. Finally, IL-4 was associated with DLco ≤ 60% of the predicted and with fibrotic involvement of ≥ 10% of lung parenchyma in SSc patients.

In our study, we demonstrated that serum level of Th2 interleukin and chemokine are increased in SSc patients than HC. In SSc pathogenesis, the role of Th2 inflammation is misunderstood. The role of inflammation, especially Th2 inflammation, in the SSc ILD has long been debated [23, 24]. Th2 cytokines, released in early ILD inflammatory phase, lead to the activation of alternative inflammatory pathways and to the transcription of TGF-β, involved in induction and progression of fibrosis [9]. According to previous studies [11, 25], we demonstrated increased Th2 cytokines serum levels in SSc patients compared to HC. Moreover, and more importantly, we found a positive linear correlation between the percentage of ground glass on HRCT and Th2 cytokines serum levels (IL-4, IL-31, IL-21, IL-13 and IL-5) in SSc patients. IL-21 seems to stimulate a Th2 dependent fibrotic response by acting as a trigger on IL-4 and IL-13, indeed it has been shown that there is an association between IL-4 and IL-21/IL-21R mRNAs, suggesting that IL-21 can promote fibrosis by facilitating the development of a Th2 response [26]. It is well known that IL-4/IL-13 axis upregulate genes known to be involved in the mechanisms of wound healing and fibrosis in murine models and in vitro [10]. IL-13 is required for an effective Th2 immune response and appears to have a direct profibrotic function [24]. IL-13 promotes epithelial metaplasia and remodeling of the airways by acting on the same receptor as IL-4, acting directly on the production of TGF-β or stimulating its signaling mediated by Smad [10]. Several studies suggested that Th2 cytokines contributed to the SSc pathogenesis and it is well known that some of these cytokines play a key role in the differentiation and migration of eosinophils, which are involved in inflammatory process in SSc patients [13]. IL-4 and IL-13 promote the secretion of IL-5 to activate eosinophils inducing fibroblast proliferation and promoting the expression of TGF-β and the epithelial-mesenchymal transition [27]. There is evidence that Th2 cells play important roles during the inflammatory/maintenance phase of pulmonary fibrosis. Th2 cytokines IL-4, IL-5, and IL-13 have been causally linked to the development of fibrosis in several chronic inflammatory diseases. Overexpression of IL-4 or IL-13 in the lung of murine models showed a profibrotic role of both cytokines influencing the activation of myofibroblasts [10]. IL-5 can also promote fibrosis in the lung by recruiting eosinophils that produce TGF-β, platelet derived growth factor (PDGF), and IL-13.

It is well known the correlation between early ILD inflammatory phase and ground glass pattern on HRCT [28]. In this study, for the first time, we demonstrated a correlation between increased Th2 cytokine serum levels and the extent of ground glass pattern on HRCT. We may suppose that Th2 cytokine are involved in pulmonary inflammation in SSc patients and that have a pivotal role in damage repair mechanisms. Afterwards, Th2 cytokines are involved in induction and maintenance of fibrotic pathways (i.e. Smad) that lead to the activation of TGF-β, PDGF and IL-6 [28]. Increased expression of genes regulated by TGF-β has been confirmed in patients with progressive lung fibrosis. In our study, reticular and honeycombing pattern showed no correlation or showed a negative linear correlation with serum IL-4. Therefore, we may suppose that in the advanced stages of ILD profibrotic cytokines prevail, such as IL-6 and TGF-β, and Th2 cytokines are less secreted.

Ando et al. [29] demonstrated higher circulating eosinophils in SSc patients compared to patients with other systemic autoimmune diseases and they found an association between eosinophils count and severity of radiological ILD only in SSc patients. In our study we demonstrated a positive linear correlation between circulating eosinophil count and ground glass pattern measured by CALIPER software.

We also found a negative linear correlation between DLco and IL-4, IL-5, IL-31 and eosinophils. Moreover, in logistic regression analysis, IL-4 was the only variable associated to DLco ≤ 60% of the predicted. A previous study found a negative correlation between elevated IL-31 serum levels and DLco [25]. Previous studies demonstrated a negative linear correlation between eosinophils count in bronchoalveolar lavage fluid (BAL) and DLco [30, 31]. These findings support the role of Th2 inflammation in early stages of ILD characterized by a reduction of DLco with preserved FVC.

This study has some limitations: the monocentric design, the lack of data about Th2 cytokines and eosinophils in BAL and absence of fractional exhaled nitric oxide (FeNO) test.

## Conclusions

We can conclude that serum level of Th2 cytokines were higher in SSc patients than HC. Th2 cytokines might have a key role in early phase of ILD in SSc patients and IL-4 was associated with DLco ≤ 60% of the predicted and with fibrotic involvement of ≥ 10% of lung parenchyma in SSc patients. Future large studies are needed to confirm these findings.

## Abbreviations

ACR/EULAR: American College of Rheumatology/European League Against Rheumatism Collaborative Criteria
BAL: Bronchoalveolar lavage fluid
CALIPER: Computer-Aided Lung Informatics for Pathology Evaluation and Ratings
DAI: Ddisease activity inde
dcSSc: Diffuse cutaneous systemic sclerosis
DLco: Diffusion lung capacity for carbon monoxide
DSS: Disease severity scale
FeNO: Fractional exhaled nitric oxide
FEV1: Forced expiratory volume in the 1st second
FVC: Forced vital capacity
HC: Healthy controls
HRCT: High resolution computed tomography
ILD: Interstitial lung disease
IQR: Interquartile range
lcSSc: Limited cutaneous systemic sclerosis
mRSS: Modified Rodnan skin score
NVC: Nailfold videocapillaroscopy
OR: Odds ratio
PAH: Pulmonary arterial hypertension
PDGF: Platelet derived growth factor
PFTs: Pulmonary function tests
SSc: Systemic sclerosis
TGF: Transforming growth factor
